# Concerned friends of intimate partner violence survivors: results from the myPlan randomized controlled trial on college campuses

**DOI:** 10.1186/s12889-023-15918-y

**Published:** 2023-05-31

**Authors:** Tina L. Bloom, Nancy Perrin, Megan Lindsay Brown, Jacquelyn Campbell, Amber Clough, Karen Trister Grace, Kathryn Laughon, Jill Messing, Karen B. Eden, Rachael Turner, Nancy Glass

**Affiliations:** 1 School of Nursing, Notre Dame of Maryland University, Baltimore, Maryland US; 2 School of Nursing, Johns Hopkins University, Baltimore, Maryland US; 3 School of Social Work, Arizona State University, Tempe, Arizona US; 4 School of Nursing, George Mason University, Virginia, US; 5 School of Nursing, University of Virginia, Charlottesville, Virginia US; 6 Dept of Medical Informatics & Clinical Epidemiology, Oregon Health & Science University, Portland, Oregon US

**Keywords:** Decision support techniques, IPV, Dating violence, Bystander intervention for IPV survivors

## Abstract

**Background:**

Nearly half of intimate partner violence (IPV) survivors experience their first abusive relationship at college age (18–24 years). Most often they disclose the violence to friends. Existing college campus “bystander” interventions training peers to safely intervene have been effective in sexual assault prevention; similar interventions have rarely been tested for IPV. Therefore, we evaluated the effectiveness of an interactive, personalized safety decision and planning tool, myPlan app, on decisional conflict, decisional preparedness, confidence in intervening, supportive safety behaviors, and IPV attitudes with concerned friends of abused college women.

**Methods:**

We recruited college students (age 18–24, *N* = 293) of any gender who had a female-identified friend who had recently experienced IPV (“concerned friends”) from 41 Oregon and Maryland colleges/universities. Participants were randomized to myPlan (*n* = 147) or control (usual web-based resources; *n* = 146). Outcomes included decisional conflict, decisional preparedness, confidence to intervene, safety/support behaviors, and IPV attitudes.

**Results:**

At baseline, concerned friends described the abused person as a close/best friend (79.1%); 93.7% had tried at least one strategy to help. Most (89.2%) reported concerns their friend would be seriously hurt by the abuser; 22.7% reported extreme concern. Intervention participants had greater improvements in decisional conflict (specifically, understanding of their own values around the decision to intervene and help a friend) and decisional preparedness immediately after their first use of myPlan, and a significantly greater increase in confidence to talk with someone about their own relationship concerns at 12 months. At 12-month follow-up, both intervention and control groups reported increased confidence to intervene, and did not differ significantly in terms of percentage of safety/support strategies used, whether strategies were helpful, or IPV attitudes.

**Conclusions:**

A technology-based intervention, myPlan, was effective in reducing one aspect of decisional conflict (improving clarity of values to intervene) and increasing decisional preparedness to support a friend in an unsafe relationship. Information on IPV and related safety strategies delivered through the myPlan app or usual web-based resources both increased confidence to intervene with a friend. College students in the myPlan group were more likely to talk with someone about concerns about their own relationship, demonstrating potential for IPV prevention or early intervention.

**Trial registration:**

Clinicaltrials.gov ID: NCT02236663, registration date 10/09/2014.

## Background

Nearly half of intimate partner violence (IPV) survivors experience their first abusive relationship at college age (i.e., 18–24 years). In research studies, between 10 and 65% of US college students report experiencing violence from an intimate/dating partner or ex-partner [1–4], a wide range likely attributable to varying operational definitions, populations, and samples. IPV among woman-identified college students has been associated with serious consequences to health and well-being, including traumatic brain injury, depression, anxiety, post-traumatic stress, somatization symptoms, increased use of alcohol, exposure to a partner controlling contraception and difficulty negotiating condom use [2, 5–8].

To reduce violence exposures and their negative impacts, IPV survivors need access to advocacy, evidence-based risk assessment tools, and safety planning information specific to their unique situation [9], most commonly accessible by connecting with trained IPV advocates via formal service providers (e.g., crisis services, campus women’s centers, healthcare, campus security and law enforcement). However, most survivors seek help from friends/family first; many never engage with formal services [10–14]. This has been shown in research with college students who experience dating and sexual violence as well [5, 15, 16], due to stigma, fear, embarrassment, privacy concerns, minimization or denial, or other concerns [16, 17]. For example, only 4.1% of undergraduate women sexually assaulted during college in a large web-based study [18] reported the assault to police; 8.4% sought help from crisis services or healthcare providers. Conversely, two-thirds (67.1%) told a friend or family member about the assault. College students commonly report witnessing dating violence, observing the effects upon their friends, and/or being the recipient of disclosures of abuse [19].

Witnessing or being recipients of IPV disclosures may have significant “ripple effects” on the members of a survivor’s social network [3, 20]. In the context of dating violence, peers in a survivor’s social network may be entangled in their own complex relationships with both parties – they may be part of the same close friend group/group dates or have introduced the couple or facilitated the relationship; they may be helpful when relationship conflicts arise but may also indirectly or even actively be the cause of relationship conflicts themselves [21]. They also must navigate their own internalized lessons from families and communities about gender, responding to IPV, whether to get involved in “private matters”, and formal help-seeking [17, 22].

The limited research conducted with IPV survivors’ friends demonstrates that they may feel positively about their actions to try to help their friends, but also may experience discomfort, anger, fear, frustration, and concerns about making the situation worse; they often feel unprepared to help [19, 23–26]. College students may lack knowledge and skills to respond to IPV and may endorse IPV myths (e.g., alcohol causes a partner to use violence), respond in unhelpful or harmful ways to disclosures (e.g., victim-blaming), and they often lack awareness of available campus and community resources [4, 17, 24].

Popular approaches to violence prevention engaging bystanders, or peers/witnesses, have been deployed on college campuses with the aim of changing social norms around sexual and dating violence, promoting prosocial (helping) behaviors, and equipping bystanders with skills to recognize and intervene in situations where violence may occur (e.g., Mentors in Violence Prevention, Green Dot, InterACT, RealConsent). However, with rare exceptions [27, 28] these are focused heavily or exclusively on primary prevention of sexual assault. Additionally, they often include community-level scenarios in which the victim, perpetrator, and/or ‘‘bystander’’ are potentially unknown to each other, limiting their utility in helping friends navigate the complexities of dating violence. Overall, the efficacy of bystander interventions in dating violence prevention has yet to be established [28, 29].

More research is needed to understand how to effectively support members of survivors’ social networks to raise awareness, increase knowledge, and build skills to provide tangible and emotional support to their friends who are navigating IPV. Thus, the purpose of the current study was to describe the impact of myPlan, a tailored mHealth safety decision aid, on concerned friends (college students of any gender, ages 18–24) self-identified as having a female-identified friend that had recently (past 6 months) experienced IPV.

myPlan is free, secure and accessible via mobile download (Apple Store, Google Play) or web browser (myPlanApp.org) [30, 31]. Decision aids are tools to support decision-making for people faced with complicated choices, particularly where there is not a clear right or wrong answer, and when understanding their own values, priorities, options, and resources is supportive (e.g., end of life decisions, prevention and treatment course for chronic illness, safety in abusive relationships) [32, 33]. myPlan’s development was grounded in Dutton’s foundational empowerment work [34], survivor and advocate input and expertise [35–37] safety planning literature [9, 38, 39] and a previous trial of an internet safety decision aid for woman-identified participants of all ages [31, 33].

myPlan addresses three key factors: [1] protection, a focus on increasing safety; [2] enhancing decision-making around safety; and [3] reducing IPV to facilitate healing. myPlan is highly interactive; it educates users about IPV dynamics and myths, and assists users to assess relationship health and safety factors, consider their priorities in decision-making, learn about available resources, and design a safety plan tailored to their individual needs and priorities, with embedded links to connect directly to resources such as advocacy, mental health, and reproductive health services [30]. myPlan has been shown highly effective for supporting survivors’ decision-making and safety [7, 31, 33, 35]. For 18–24-year-old woman-identified college students experiencing IPV, myPlan significantly reduced reproductive coercion and risk for suicide compared to usual web-based resources [7]. However, myPlan’s effectiveness to provide concerned friends with the information and resources to support their friend in an unsafe relationship has not been previously examined. We hypothesized that over the study period, concerned friend users randomized to myPlan would endorse more informed IPV attitudes, experience more individualized decision support, and thus have less decisional conflict about helping a female friend in an abusive relationship, increased confidence in their ability to do so, and would use more effective safety/support strategies.

## Methods

### Sample

The sample was drawn from 41 diverse college/universities, including community colleges, in Maryland and Oregon. Participants were recruited primarily online, via extensive posting on social media, outreach through campus/student listservs and bulletins, often via campus women’s centers and other similar campus services. Eligibility criteria were: (1) English-speaking, (2) any gender, (3) age 18–24, (4) attending college or university at least part-time in Maryland or Oregon, and (5) reported having a female-identified friend that experienced IPV in the past 6 months (hereafter referred to as the “survivor”). Additionally, participants had to have access to a safe smartphone or computer with internet, safe email, and express comfort with their ability to download an app and/or use the internet.

#### Procedures

Eligible participants were automatically randomized at enrollment to intervention or control groups using an automated computerized blocked randomization algorithm, with randomization stratified by state (to ensure group proportions remained relatively constant) and college/university type (two-year technical school, community college, four-year state college/university, four-year private college/university, or other). This ensured that the proportion of friends in each state assigned to each group remained relatively constant during the study. Research assistants were not blinded to study condition. Once participants consented and were enrolled and randomized, they received an automated email at the safe email address provided at enrollment, which provided their username and password and instructed them how to access the study site. Intervention group participants could download the app or access a browser-based version from the study website. Participants entered their username and password on first use and were prompted to create a private 4-digit security PIN code for future access. The PIN could be reset by logging out and back in using their username and password.

**Fig. 1:**
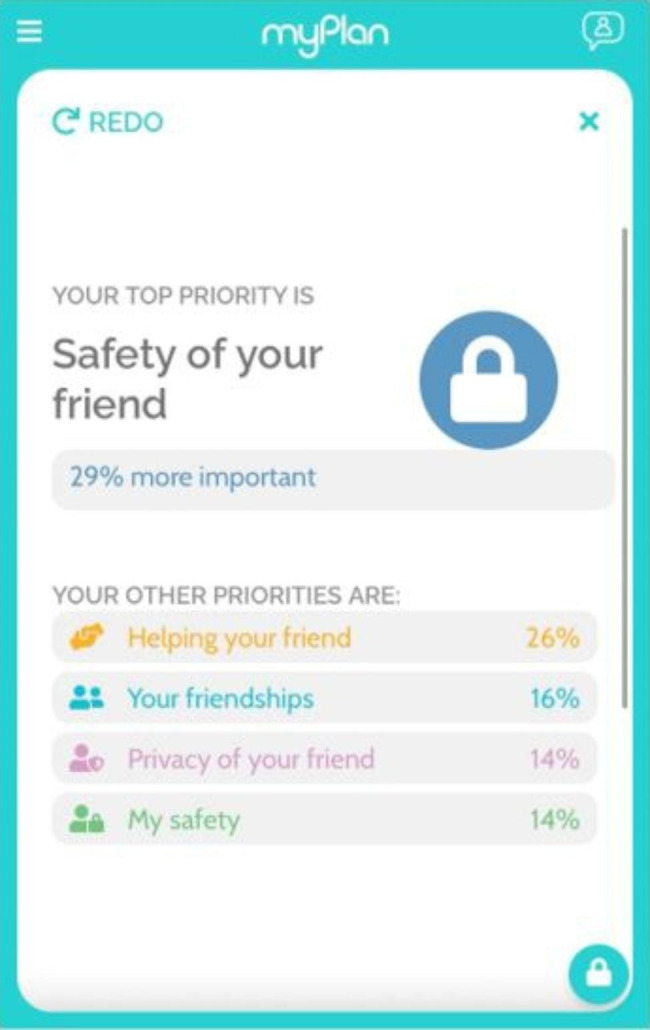
Example of feedback on priority-setting exercise

The myPlan intervention for concerned friends includes the following sections: [1] educational content regarding IPV and healthy relationships, including IPV dynamics and common myths; [2] a safety assessment section which elicited user input on their female friend’s relationship, with immediate, tailored feedback on red flags for unhealthy relationships, including the Danger Assessment (DA) or DA-Revised (for women abused by female partners), a validated, widely-used tool which identifies and provides tailored feedback on the severity of the abuse situation and risk of repeat severe IPV [9, 40]; [3] an interactive priority-setting tool that allows users to make pairwise comparisons of their priorities and values regarding concern for their friend’s safety, privacy, personal safety, and social support/status and receive feedback (see Fig. 1); [4] a safety plan with strategies and resources to support their friend and also keep themselves safe. All participants were provided core safety strategies (e.g., how to talk with a friend about their concerns for her safety). Additional strategies were tailored to their input, for example, how to support a friend still in the relationship (based on input regarding survivor plans for the relationship); managing safety on campus (if the abusive partner/ex is a student at the same college); or how to find LGBTQ-specific community resources for support (based on input regarding the survivor’s relationship demographics).

Control group participants accessed safety planning via a secure study website and received basic emergency safety planning strategies to support a friend, and where to find information or help on campus or via websites geared toward college students. This “usual care” approach was not individualized (e.g., it did not provide tailored feedback or interactive sections, or generate a plan with tailored strategies based on input). Rather, the static emergency safety plan was the same for all users; it provided two resources (national IPV hotline and national LGBTQ hotline) and did not provide comprehensive safety information or resources beyond emergency safety planning. A detailed description of the approach for both groups is provided in the published study protocol [30].

Participants completed all data collection via the secure app/website, which allowed them to complete surveys at their convenience, helped reduce data input error, and potentially reduced social desirability bias compared to face-to-face interviews [41, 42]. Both groups could access the study websites/app as much as they liked during the one-year follow-up period. Participants were prompted via their safe contact information to complete follow-up assessments with a total time burden of ~ 3 h over the study duration. Participants received gift cards for their time and expertise (baseline $20, 6-month $30, 12-month $50; total $100). The Johns Hopkins Medicine Institutional Review Board (IRB 00054334) and the Kaiser Permanente Center for Health Research IRB (Pro00004875) approved human subjects procedures. Study and safety procedures are detailed in the protocol publication [30].

#### Measures

**Descriptive measures**. We collected demographics on the concerned friend, female survivor and her abusive partner as described by the concerned friend (age, gender, race/ethnicity, children in home, year in college/university, type of institution, student enrollment status, employment). We also asked concerned friends to report on characteristics of survivors’ abusive relationship status (e.g., current or ex-partner, marital status, relationship duration, living situation of survivor/partner, whether survivor/partner enrolled at the same college). Participants were asked if they had heard about or witnessed/experienced behavior(s) by the abusive partner in ways that concerned them, if the survivor had disclosed things that concerned them, if they had asked/confronted the survivor about her partner’s/ex’s behavior(s), and how concerned they were that the survivor’s partner/ex would seriously hurt her (e.g., not concerned, a little concerned, somewhat concerned, extremely concerned). Concerned friends were asked if they had themselves experienced negative consequences (e.g., threats from the partner, physical violence from the partner, partner wouldn’t allow survivor to talk to/see them anymore, survivor didn’t want to talk to/seem them anymore) for trying to intervene and support the survivor. Lastly, concerned friends were asked about their own violence exposures (i.e., if they had personally experienced IPV either as a victim or a perpetrator, witnessed violence or abuse between parents/caregivers, or been abused as a child).

Outcomes included the following:

**Decisional conflict.** We measured concerned friends’ decision process with questions adapted from validated subscales of the Decisional Conflict Scale (DCS) [43]. The 12 items measure the concerned friends’ understanding of potential advantages and disadvantages of different strategies for supporting the survivor, and their values related to those options (e.g., “I know my options for helping my friend who is in an unhealthy relationship,” “I am clear about which reasons for helping my friend are most important to me,” “I have enough advice to make decisions about helping my friend who is in an unhealthy relationship.”). Each question has three response options (yes, no, and unsure). The DCS provides a total score as a measure of the decision process, as well as scores for four subscales ( *feeling informed, certainty about decision, values clarity*, and *support*). Lower DCS scores indicate less decisional conflict (and thus a better decision process). This measure was administered at baseline and immediately after first use of myPlan/control app.

**Decisional preparedness.** The “Preparation for Decision Making” [44] scale measures concerned friends’ perceptions regarding the usefulness of the app/website (intervention or control) and the information and resources provided to support their decisional preparedness to support the survivor. Ten questions on a 5-point scale (“not at all, a little, somewhat, quite a bit, a great deal”) were asked in response to the question stem “did the information provided about talking to your friend about safety and resources…” Example questions include: “help you think about how involved you want to be in making decisions about your friend’s safety,” “prepare you to make better decisions about your friend’s safety,” “help you organize your own thoughts about your friend’s safety,” “help you identify questions you want to ask a trusted person.” This measure was administered at baseline (after first use of myPlan/control website) and at 12 months, with total scores summed and higher scores indicating greater decisional preparedness.

**Confidence to intervene.** Confidence to intervene on behalf of a friend in an unsafe relationship was measured with an adaptation of the Self-Efficacy to Deal with Violence Scale [45]. The 19-item measure assessed how confident the concerned friend felt to address relationship issues on a 4-point scale (“not at all confident, somewhat confident, confident, very confident”) in five domains (*provide help while staying safe, recognize the signs of IPV, help with resources, provide unconditional support, talk with someone about concerns in their own relationship*). Example items include: “How confident are you in your ability to… start a conversation with a friend regarding worrisome behaviors by her partner/ex-partner”, “…identify appropriate/useful resources/people on your campus to help a friend in an unhealthy or unsafe relationship?”. This measure was administered at baseline and 12 months.

**Supportive safety behaviors**. The Supportive Behaviors Checklist assessed 23 different supportive behaviors used to assist a friend experiencing IPV. These included *social support behaviors* (e.g., “Listened patiently to your friend’s relationship concerns,” “kept in regular contact with your friend”), *formal service use* (e.g., “Met/called/texted/instant messaged a professional that works on campus – professor, administrator, staff at a women’s center or health clinic – to talk privately about concerns about your friend’s relationship”), and *safety planning strategies* (e.g., “Helped your friend pack an emergency bag or made copies of keys/kept these for her”). We adapted this measure from the Safety Behavior Checklist [46, 47] which measures the range of strategies used by IPV survivors to increase safety or escape violence. Our adaptation for friends was based on family/friend responses as described by survivors in extant literature, as well as engagement behaviors by informal social network members of IPV survivors as described in Latta & Goodman’s grounded theory model [26] and Beeble et al.,’s Approaches to Helping measure [48]. At baseline, we asked the concerned friend about whether they had used each strategy in the past 6 months and if so, “if you thought it was helpful for your friend,” with response options being “No,” “Yes, but I didn’t think it was helpful,” and “Yes, and I thought it was helpful.” Items were re-administered at 12 months with the referent time period being the previous 12 months.

**IPV Attitudes.** This construct was measured at baseline and 12 months with three scales. The Acceptance of General Dating Violence Scale [49] is a five-item scale (e.g., “There are times in a dating/intimate relationship when violence is okay,” “Someone who makes their partner jealous on purpose deserves to be hit”) with responses on a 4-point Likert scale from strongly disagree to strongly agree. Endorsement of IPV myths was measured with 5 items developed by the research team (e.g., “Leaving an abusive partner will stop the abuse,” “women can’t be abused by a female partner”) with responses on a 4-point Likert scale from strongly disagree to strongly agree. Finally, IPV awareness and perceptions of its seriousness was measured with 2 items developed by the research team, adapted from items from Beeble, Post, Bybee, & Sullivan [48]. The items are: “In your opinion, how *common* are abusive dating/intimate relationships in college?” (response options: “very uncommon, uncommon, happens sometimes, common, very common”) and “In your opinion, how *potentially serious* (damaging, harmful, or dangerous) are abusive relationships in college?” (response options: “no potential for danger or harm, generally does not cause much harm, can have some harmful effects, have harmful effects but generally not serious, potentially very serious or dangerous”).

### Statistical analysis

T-tests and chi-square tests were used to determine if randomization achieved balance between the two arms. Generalized estimating equations (GEE) with an unstructured covariance matrix and Gaussian distribution examined differences in change over time (baseline, immediate post intervention/usual care and 12-months) between the two groups. There was little attrition over time (5 people for 98.3% retention) so no replacement of missing data was used. GEE does not require complete data at all time points, so all observations are included in the analyses. In this manuscript, we are reporting on 12-month follow-up data.

## Results

***Characteristics of concerned friends***. Data were collected from 7/2015 to 10/2017. At baseline, participants (*N* = 293) were randomized to intervention (n = 146) or control (n = 147) groups. Retention was high across 12 month follow up data collection periods; in the final sample only one person was lost to follow up in the control group and 4 in the intervention group (99.3% and 97.2% retention, respectively). Table 1 summarizes the characteristics of the sample. About three-quarters (77.6%) identified as female, with a mean age 20.5 years (*SD* 1.8). Concerned friends could select multiple races; they reported their race as white (56.2%), Black (17.8%), Asian (18.8%), More than one race (9.2%) and ethnicity as Latino/Hispanic (16.4%). The only significant baseline demographic difference between groups was that the control group had more participants identifying as white than the intervention group (62.8% vs. 49.3%, respectively; *p* = .02). Few (3.8%) were in graduate school, with the remainder split nearly equally across freshman (20.4%), sophomore (30.8%), junior (20.8%), or senior (24.2%) years of undergraduate education. Participants were enrolled in two-year technical college or community college (18.3%), four-year state (37.3%) and private (44.4%) colleges and universities. Just under half reported living on campus (42.6%).

**Table 1:** Baseline Characteristics of Control Group and Intervention Group Participants

	Control N = 147 N (%)	Intervention N = 146 N (%)	Total N = 293 N (%)	p-value*
**Participant “Concerned Friend” Characteristics**				
Woman	115 (78.8)	110 (76.4)	225 (77.6)	0.883
*Age (mean, SD)*	*20.36 (1.67)*	*20.54 (1.87)*	*20.45 (1.77)*	*0.414*
Race/ethnicity				
White	93 (62.8)	71 (49.3)	164 (56.2)	0.020
Black	29 (19.6)	23 (16.0)	52 (17.8)	0.419
Asian	26 (17.6)	29 (20.1)	55 (18.8)	0.574
More than one race	18 (12.6)	9 (6.9)	27 (9.2)	0.113
Hispanic	26 (17.6)	22 (15.3)	48 (16.4)	0.616
College Status				0.225
Freshman	29 (19.9)	30 (21.0)	59 (20.4)	
Sophomore	45 (30.8)	44 (30.8)	89 (30.8)	
Junior	27 (18.5)	33 (23.1)	60 (20.8)	
Senior	42 (28.8)	28 (19.6)	70 (24.2)	
Graduate Student	3 (2.1)	8 (5.6)	11 (3.8)	
Type of College				0.933
Two-year Technical or CC	24 (18.1)	25 (18.5)	49 (18.3)	
Four-year state college or university	51 (38.3)	49 (36.3)	100 (37.3)	
Four-year private college or university	58 (43.6)	61 (45.2)	119 (44.4)	
Living on campus	53 (40.4)	58 (45.0)	111 (42.6)	0.755
**IPV Exposures**				
Experienced IPV from a boyfriend/girlfriend	46 (31.1)	41 (28.5)	87 (29.8)	0.626
Abusive to a boyfriend/girlfriend	11 (7.4)	8 (5.6)	19 (6.5)	0.516
Witness IPV between parents/caregivers	50 (33.8)	65 (45.1)	115 (39.4)	0.047
Abused by a parent/caregiver as a child	20 (13.5)	25 (17.4)	45 (15.4)	0.363
**Characteristics of Woman Friend in Unsafe Relationship**				
Type of Friend				0.145
Close Friend	55 (37.9)	73 (51.4)	128 (44.6)	
Best Friend	55 (37.9)	44 (31.0)	99 (34.5)	
Casual/other Friend	35 (24.1)	25 (17.6)	60 (20.9)	
*Age (mean, SD)*	*20.36 (2.07)*	*20.99 (3.03)*	*20.67 (2.60)*	*0.045*
Relationship status				0.357
Boyfriend/girlfriend	60 (41.1)	53 (37.1)	113 (39.1)	
Ex-boyfriend/girlfriend	59 (40.4)	64 (44.8)	123 (42.6)	
Casual/hookup/friends with benefits	17 (11.6)	11 (7.7)	28 (9.7)	
Other/Don’t know	10 (5.4)	15 (10.5)	25 (87)	
Lives with partner or spends most nights with them	46 (31.7)	53 (37.1)	96 (33.3)	0.232

*Based on t-test or chi-square

***Participants’ lifetime experiences of violence***. Nearly one in three (29.6%) concerned friends reported that they themselves are IPV survivors, and 6.5% disclosed they themselves had been abusive toward a partner (Table 1). About 1 in 7 reported abuse by a parent or caregiver during childhood, and 39.4% had witnessed parental IPV, with the intervention group significantly more likely to report witnessing IPV between parents or caregivers than the control group (45.1% vs. 33.3%, respectively; *p* = .04).

***Characteristics of survivors’ abusive relationship***. The majority of concerned friends described the survivor as a close or best friend (79.1%); 20.9% were a casual friend/classmate (Table 1). The mean age of survivors was 20.7 years (*SD 2.6*), with a slight but statistically significant difference in age between control (20.4; *2.0*) and intervention (20.9; *2.60 p* = .04) groups. In 39.1% of cases, the abuser was the survivor’s current partner; in 42.6% it was an ex-partner. One-third of survivors (33.4%) either lived with the abuser or spent most nights with them. Slightly over half (51.0%) of concerned friends had personally witnessed concerning behaviors by the survivor’s partner/ex-partner, 55.0% had heard from others about concerning behaviors, and 50.7% had talked to the survivor about their partner/ex’s behavior. The majority (80.5%) reported the survivor had shared things about her relationship that concerned them. Most friends (89.2%) reported concern for the survivor’s safety, believing the partner or ex- could physically and/or sexually hurt her, with 22.7% reporting extreme concern. Concerned friends commonly reported experiencing negative consequences when trying to help the survivor, including experiencing threats (13.4%) or actual physical violence (3.8%) from the abusive partner. About one-quarter (23.6%) became cut off from the survivor as the abusive partner would not let the survivor see or talk to the concerned friend, and 17.8% of friends reported survivors no longer wanted to see or talk to them after they expressed their concerns about the relationship.

***Changes in concerned friends’ decisional conflict, decisional preparedness, and confidence to intervene over time***. Concerned friends in the intervention group had a greater reduction in decisional conflict in one of the subscales of the DCS immediately following use of myPlan than concerned friends in the control group. Specifically, they had improved clarity regarding their own values about providing information and support to their friend (*p* = .047; Table 2). In terms of decisional preparedness, the intervention group had higher scores than the control group after using the app/website one time (M = 3.85 vs. 3.58, p = .002). This trend continued at 12 months, although the difference was not statistically significant (M = 3.89 vs. 4.05; p = .068). At baseline, both groups reported high levels of confidence to help a friend (i.e., close to a 3 on 4-point scale), provide help while staying safe, recognize the signs of IPV, help with resources, provide unconditional support, and talk with someone about concerns in their own relationship, with the highest being confidence in their ability to provide unconditional support. Both groups increased over time on each item; confidence to “talk with someone about concerns about my own relationship” was significantly higher in the intervention than the control group at 12 months (*p* = .035; Table 3).

**Table 2:** Changes in decisional conflict subscales, pre and post intervention/control tool use

Subscale	Control Group Mean Score		Intervention Group Mean Score		Interaction p-value*
	Pre	Post	Pre	Post	
Feeling Informed	40.86	31.79	40.44	31.02	0.837
Certainty About Decision	50.89	41.74	48.01	38.40	0.720
**Values Clarity**	**36.76**	**32.79**	**36.71**	**30.05**	**0.047**
Support	44.00	36.46	44.10	35.51	0.602
Total Scale	43.14	35.68	42.31	33.74	0.265

**Table 3:** Changes over time in domains of confidence to intervene on behalf of a friend

Domain	Control Group Mean Item Score		Intervention Group Mean Item Score		Interaction *p*-value*	
	Baseline	12 months	Baseline	12 months		
Provide help while staying safe	2.78	3.05	2.77	3.09	0.148	
Recognize signs of IPV	2.77	2.93	2.67	2.92	0.268	
Help with resources	2.49	2.88	2.52	2.97	0.835	
Unconditional support	3.04	3.17	3.03	3.16	0.344	
Talk to someone about concerns in own relationship	**2.70**	**2.99**	**2.69**	**3.04**	**0.035**	
Total Scale	2.75	2.98	2.72	3.01	0.265	

***Changes in strategies to help their friend over time***. At baseline, 93.7% had already tried at least one strategy to help their friend, with social support strategies the most commonly used type of strategy. At baseline, the intervention group reported 70.0% of the strategies they had already tried were helpful to the survivor; the control group reported 71.2% of strategies they tried as helpful. At 12 months, when participants were asked to report on the perceived helpfulness of strategies used in the past 12 months, the two groups did not report change in helpfulness of strategies over time (69.1% intervention and 70.6% control; p = .103). The pattern was consistent across type of strategies, including social support strategies (70.8% control vs. 69.1% intervention at baseline; 72.1% control vs. 70.1% intervention at 12-months), formal service use (63.1% control vs. 57.7% intervention at baseline; 71.0% control vs. 67.2% intervention at 12-months), and safety planning strategies (74.3% control vs. 75.7% intervention at baseline; 77.6% control vs. 80.1% intervention at 12-months); there were no differences in the pattern of change over time between the two groups.

## Discussion

To our knowledge, no previous study has examined the effectiveness of a technology-based safety decision and planning aid to promote concerned friends’ use of supportive safety behaviors with a female friend in an abusive relationship, even though evidence indicates friends are likely to witness abusive behaviors and/or be recipients of IPV disclosure. We successfully recruited a convenience sample of 18–24-year-old students on 41 college/university campuses in Maryland and Oregon who were concerned about a female-identified friend in an unsafe relationship with a current or ex-partner and retained 98.2% of them over one year, randomizing them to the tailored myPlan app or to a static (non-tailored) study website with basic emergency safety planning strategies to support a friend, where to find campus or internet-based information or help, and two resources (a national IPV hotline and national LGBTQ hotline). In comparison to the control condition, after one use of myPlan, we found that users had significantly reduced decisional conflict in terms of increasing clarity of values for intervening, and significantly increased decisional preparedness. At 12 months, myPlan users continued to have greater decisional preparedness than control users, but the difference was no longer statistically significant at < 0.05, although still large (*p* < .068). Both groups were highly confident in their ability to intervene on behalf of an abused friend at baseline and were similar at baseline and 12 months follow-up in the percentage of support and safety strategies used, the percentage of used safety strategies perceived as helpful to the survivor, and in their attitudes toward IPV. At 12-month follow-up, both groups reported even greater confidence to intervene on behalf of a friend, with no difference in significance between the groups except on one item -- myPlan users had a significantly greater increase in confidence to talk with someone about concerns in their own relationships.

After one use of myPlan, we found the intervention group had a greater reduction in one of the components of decisional conflict; they reported greater clarity regarding their own values *p* = .047). The intervention group also reported higher scores on decisional preparedness (M = 3.85 vs. 3.58, p = .002) wherein the information provided about talking to their friend about safety and resources helped participants think about their level of involvement in their friends’ decision-making, prepare to make better decisions, organize their own thoughts about their friend’s safety, and identify important questions. The trend regarding higher scores on decisional preparedness continued at 12 months, approaching but not meeting statistical significance (M = 3.89 vs. 4.05; p = .068). Decisional conflict – essentially a state of uncertainty about a course of action when facing risky, difficult, and/or complicated decisions, can lead to decisional paralysis, with delayed decision-making and vacillation between choices [50]. Decisional conflict can be decreased when people have sufficient information about alternatives, benefits, and risks, have clarity on their personal values related to their decisions, and experience support for their decisions [43, 50]. This finding has particular salience for young adults who are often bystanders to unsafe relationship behaviors in their friend groups, with potential entanglement or complex relationships or friendships with both partners [21] and their own internalized lessons learned about gender, violence, privacy, and formal help-seeking [17, 22]. Social networks are the most frequently-accessed resource for survivors [5, 15, 16]. Thus, clarifying personal values and reducing decisional uncertainty among friends is likely valuable for creating effective support, prosocial behaviors, and reducing the substantial impact of partner violence on college students.

Both intervention and control concerned friends started off relatively high at baseline (nearly 3 on a 4-point scale) in terms of confidence to help a friend in an abusive relationship (e.g., to provide help while staying safe, recognize the signs of IPV, help with resources, talk with someone about concerns in their own relationship, and in particular, to provide unconditional support). The high baseline levels of confidence may reflect the non-representativeness of our sample; compared to the typical college student, those who responded to recruitment materials and enrolled in this study had already recognized abuse and nearly all had actively intervened with at least one strategy. Over time, both groups also increased on every item related to confidence in their ability to intervene, but the intervention group had a significantly greater increase in confidence on the item regarding their ability to talk with someone regarding concerns in their own relationships. Research on survivors’ help-seeking behavior has thoroughly documented that few survivors access formal services; a large percentage may never disclose IPV to anyone at all [10–14]. This intervention was intentionally designed to bridge this gap, not by replacing advocacy services, but by providing users the information, resources, feedback, and support to consider their own safety priorities, build a safety plan and a support network, and learn about and connect to existing services [35]. For this reason, its psychoeducation content and safety planning strategies both heavily emphasize connecting with trusted others for support and education about available campus and community resources, which may help explain this particular finding. Additionally, for concerned friends using myPlan, they may have increased self-awareness regarding their own relationship, with the information and resources provided in myPlan allowing them the knowledge and space to reflect on concerning or abusive behaviors in their current or past intimate relationship. This is potentially an important intervention strategy given the high prevalence of IPV among this age group [1, 2] as safety strategies tailored for supporting a friend increased their own awareness of, and comfort in, accessing confidential and supportive services in their campus or community. Further, this provides a potentially important avenue for intervention – among college-age people, focusing intervention strategies on helping friend networks may result in broader reach, greater awareness of abusive relationship behaviors, and engagement in intervention.

Descriptive baseline findings underscore that – consistent with extant research with college students [1, 2] -- our participants experienced high IPV exposures in their own lives, as well as significant consequences as bystanders to IPV in their friends’ relationships. Nearly one in three reported being abused by a partner, about 1 in 15 reported perpetrating IPV, and > 1 in 3 had witnessed IPV between their parents/caregivers. Most described the abusive relationship that they were concerned about as affecting a close or best friend (79.1%) and reported they themselves had been directly affected by the abuse, including threats (13.4%) or actual physical violence (3.8%) from the abuser. A large proportion of concerned friends reported losing their friendship with the survivor because of the IPV, with 23.6% reporting the abuser would not let the survivor see or talk to them anymore, and about 17.8% reporting the survivor no longer wanted to see or talk to them after they expressed their concerns about the relationship.

Prior research has documented the impact of IPV on friends or family of the survivor; some have reported feeling good about their efforts to help, but many do not [19, 23–26]. While our participants had high levels of confidence and perceived most of their actions as helpful, many were quite worried about their friends; at baseline 89.2% reported concern about the potential for serious harm by the abuser and nearly 1 in 4 expressed extreme concern for their friend’s safety. Given the dense social networks within colleges and universities, the primary importance of friendships at this stage of a person’s life and to their health [51], and the sometimes-painful and complex entanglements of relationships involving friends and IPV [21], future researchers should consider measurement of the ongoing “ripple effects” on social network members with this population. Additionally, it is important to note that bystander interventions (where students are trained to interrupt potential instances of sexual violence, e.g., looking out for a highly intoxicated woman at a party) have been widely adopted across college campuses [52] and are often extended to include training students to intervene in IPV, where dynamics and interrelationships may be very different. Our findings suggest it is important for campus bystander and dating prevention programs to consider carefully the potential negative impacts on concerned friends when they witness abusive behaviors and when they express their concerns to the survivor and integrate safety strategies and supportive resources such as counseling.

### Limitations

Our sample was comprised of college students who had identified IPV in a friend’s relationship -- in most cases a close friend -- and many were highly worried about the survivor’s safety. It is likely our study volunteers were more attuned to and knowledgeable about IPV compared to the broader population of college students. Their confidence in their own ability to be helpful and their perceptions of the helpfulness of their own actions were high when they came into the study and remained high throughout (regardless of group assignment). It is worth noting that research (e.g., see Sylaska & Edwards’ review) [53] has found that IPV survivors report wide variations in the helpfulness of the responses they receive when they disclose IPV to people in their informal social support networks, with impact on their mental health. It may be useful for future intervention researchers to measure the helpfulness of concerned friends’ actions from the perspective of the survivors themselves. Another limitation worth noting is that while the control website was static (non-tailored), we did provide evidence-based, emergency safety planning support, information on how to find campus or internet-based information/help, and hotline information. For ethical reasons, it is important for IPV researchers to provide some level of resources to all study participants, given the potential seriousness of IPV and its sequelae. Our control website may have represented a more active condition and intervention in and of itself, accounting for outcomes on which the two study conditions exerted similar effects. In fact, this kind of carefully designed website, giving extensive information about campus as well as internet-based national resources, would ideally be offered widely at community and 4 year college campuses in addition to the myPlan app.

Finally, our total sample was predominantly undergraduate students (96.2%). While a majority of graduate students are age 25 and older (62.7% of first-year graduate students and 80.5% of those in 2nd year or higher) [54]these figures suggest graduate students in the 18 to 24 year old age range are underrepresented in our sample. This may be in part because 1 in 5 of our students (18.5%) came from community college settings – a population largely overlooked in IPV research– but also suggests that researchers who wish to include graduate students in their sample may need more deliberate outreach to that population.

## Conclusion

College students report a high IPV prevalence, which has substantial impacts on those who directly experience it as well as their social networks. Friends are frequently the primary support system for survivors and the centrality of peer relationships during this developmental period make this a particularly salient intervention point for college-age people. Thus, it is critical to find effective ways to support friends in understanding IPV and providing confidential, helpful support and safety strategies with ongoing access to campus-based trauma informed services and counseling for friends, bystanders and others who reach out to help a survivor. In this study, technology-based resources (both the safety planning app and usual safety website) increased participants’ confidence for intervening to support or help a friend in an unsafe relationship. The myPlan intervention was effective compared to the control group in clarifying concerned friends’ values for supporting their friend and recognizing and seeking support for concerns about their own intimate relationship, suggesting that technology-based interventions are useful to IPV survivors and their friends on college/university campuses.

## Data Availability

The datasets generated and/or analyzed during the current study are not publicly available due to safety considerations for intimate partner violence survivors but are available from Dr. Nancy Glass (nglass1@jhmi.edu) upon request.

## List of Abbreviations

IPV: Intimate partner violence
